# Catalytic C–H to C–M (M = Al, Mg) bond transformations with heterometallic complexes

**DOI:** 10.1039/d0sc03695a

**Published:** 2020-08-17

**Authors:** Maria Batuecas, Nikolaus Gorgas, Mark R. Crimmin

**Affiliations:** Department of Chemistry, Molecular Sciences Research Hub, Imperial College London 80 Wood Lane, Shepherds Bush London W12 0BZ UK m.crimmin@imperial.ac.uk

## Abstract

C–H functionalisation is one of the cornerstones of modern catalysis and remains a topic of contemporary interest due its high efficiency and atom-economy. Among these reactions, C–H borylation, that is the transformation of C–H to C–B bonds, has experienced a fast development because of the wide utility of organoboron reagents as synthetic intermediates. The mechanistic background is now well-understood and the role of transition metal boryl or σ-borane intermediates in this transformation is well documented. This mini-review focuses on efforts made by our group, and others, to establish palladium- and calcium-catalysed methods for C–H metalation employing heavier main group elements (M = Al, Mg). These are new catalytic reactions first accomplished in our group that we have termed C–H alumination and magnesiation respectively. Unusual heterometallic complexes have been identified as key on-cycle intermediates and their unique reactivity is discussed in the context of new catalytic pathways for C–H functionalisation. Hence, this mini-review summarises the recent progress in the area of C–H metalation reactions as well as the new opportunities that may arise from this concept.

## Abstract 2

This highlight focuses on recent efforts to establish catalytic methods for C–H functionalisation with main group metals (M = Al, Mg).

## Graphical abstract

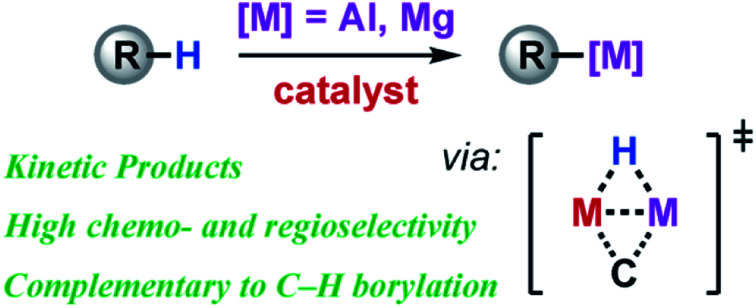

## Introduction

The transformation of simple hydrocarbons into more complex and valuable products *via* catalytic C–H bond functionalisation has revolutionised modern synthetic chemistry. This type of reactivity has quickly become one of the cornerstones of modern catalysis as it provides a straightforward and highly efficient strategy to form C–C or C–X bonds from C–H bonds (X = heteroatom). Catalytic C–H functionalisation processes have found widespread applications ranging from upgrading simple hydrocarbons to sophisticated late-stage functionalisation of complex molecules.^1^ Despite the widespread development of C–H functionalisation reactions, further improvements in scope, catalyst activity, and catalyst selectivity are still desirable.^2^ The majority of catalytic methods requires high metal catalyst loading,^3^ which limits their industrial applications. Furthermore the presence of directing groups is typically used to achieve high site selectivity.^4^ To address these limitations, a number of new base-metal catalysts^5–7^ and new approaches to selectivity control, *e.g.* through non-covalent interactions, have recently emerged.^8–10^

Particular progress has been achieved in the field of transition metal (TM) catalysed C–H borylation, that is the transformation of C–H to C–B bonds. Due to the high utility of organoboron reagents as synthetic intermediates, C–H borylation is now widely employed in the construction of natural products, active pharmaceutical ingredients, and building blocks for organic polymers.^11^ A number of transition-metal^12,13^ and main-group^14–16^ catalysts have been reported for C–H borylation of a range of substrates including heteroarenes, arenes, alkenes and alkanes. The mechanistic background of transition metal catalysed C–H bond borylation has been extensively studied over the past few decades and is well-understood. Thermodynamically, the formation of C–B bonds provides a driving force in these reactions.^17^ Kinetically, C–H borylation reactions proceed *via* low energy pathways in which transition metal boryl species often play an essential role. The intrinsic properties of boryl ligands facilitate the elementary C–H activation and C–B bond formation steps. For example, the strong σ-donor ability of the boryl ligand can stabilise high oxidation state transition metal complexes necessary for redox-based catalytic cycles.^18^ On the other hand, the accessible p-orbital of the boron atom of these ligands is decisive for C–H/B–H σ-bond metathesis mechanisms occurring at redox-neutral transition metal centres.^19^

**Scheme 1:**
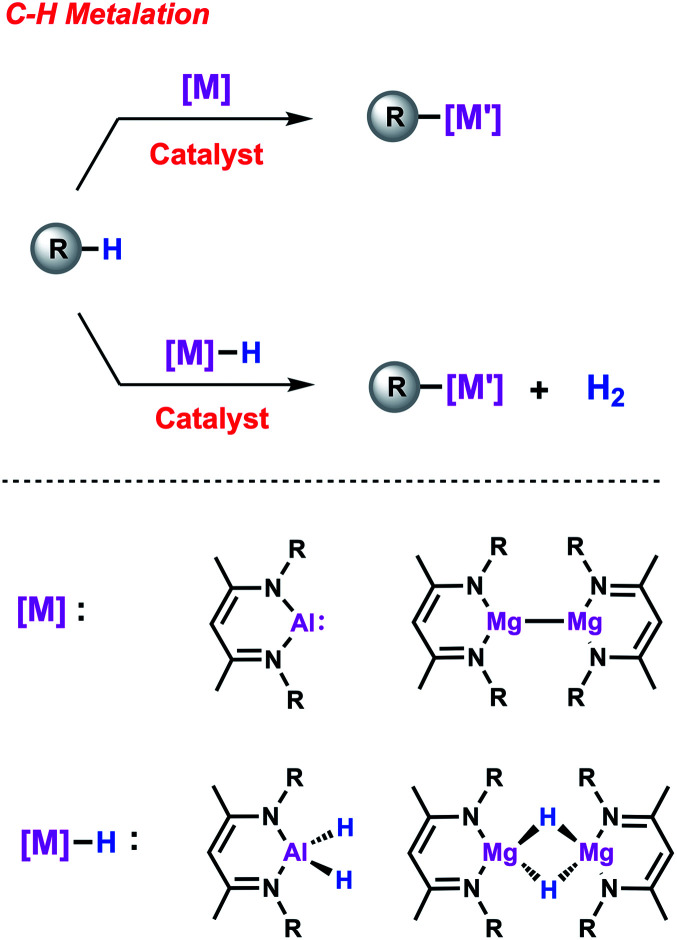
Outline of metal-catalysed C–H metalation reactions.

Beyond boron, organometallic reagents based on main group metals such as aluminium or magnesium have been an important aspect of synthetic chemistry since its beginnings.^20^ By analogy to C–H borylation, gaining access to synthetically valuable compounds *via* the direct metalation of simple hydrocarbons constitutes an attractive goal (Scheme 1). Recent efforts have focused on using main group species as stoichiometric reagents capable of breaking strong C–H, C–C and C–X bonds.^21–23^ A particular exciting aspect of this work has been the exploration of bimetallic systems in which cooperative effects between two main group metals play a role in determining reactivity.^24^ Further advancements can be expected if one could modify the scope and selectivity of these reactions with the aid of a TM catalyst.^25^ In this mini-review, we will address this new approach and show how the combined action of two metals can lead to new reaction pathways thus providing new opportunities in terms of reactivity and selectivity. Since direct access to new organometallic reagents is now available their potential synthetic utility will be also considered.

## Catalytic C–H metalation reactions

### C–H alumination

**Scheme 2:**
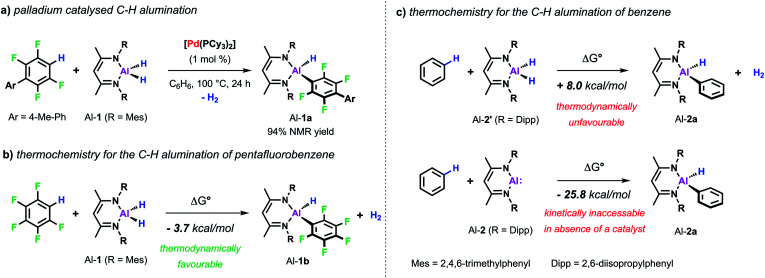
(a) Pd-catalysed C–H alumination of fluoroarenes. Calculated thermochemistry for C–H alumination of (b) pentafluorobenzene and (c) benzene, M06L-GD3 functional and hybrid (Al, SDDAll; 6-31G**, C, H, N) basis set.

Our interest in C–H metalation reactions arose from the discovery of a palladium bis(phosphine) complex as a catalyst for the dehydrocoupling of sp^2^ C–H and Al–H bonds.^26^ Reaction of fluoroarenes and heteroarenes with aluminium dihydride Al-1 in the presence of a catalytic amount of [Pd(PCy_3_)_2_] affords the corresponding metallated product in high yield. This new transformation was discovered as part of a mechanistic study on a related catalytic reaction involving the transformation of sp^2^ C–F to C–Al bonds of perfluoroarenes using [Pd(PCy_3_)_2_]. During this investigation we discovered that a two-step process was in operation, likely involving an initial hydrodefluorination of the fluoroarene, followed by C–H alumination of the resulting C–H bond. In accordance with this and with the thermochemistry calculated for the C–H alumination of pentafluorobenzene with Al-1, we found that this transformation was an extremely facile reaction and one that showed high selectivity (Scheme 2a and b).^27^ Thus when 2,3,5,6-tetrafluorobiaryls were used as substrates metalation at the single C–H position took place. Similarly, high selectivity was also observed for the C–H alumination of heteroarenes such as benzofuran, furans, or *N*-methyl indole resulting in the selective functionalisation in the 2-position of these heterocycles. When the 2-position was blocked, as in the case of 1,2-dimethylimidazole, functionalisation of the remote positions of the heterocycle by C–H alumination still took place.

These first examples demonstrating the viability of C–H alumination under catalytic conditions stimulated efforts to expand this new type of reaction to less reactive unfunctionalised aromatic substrates. However, calculations on the thermochemistry of the reaction of the aluminium dihydride Al-2′ with benzene to afford the C–Al–H product Al-2a accompanied by the liberation of dihydrogen reveal an endergonic process. The recent developments in the chemistry of main-group compounds in low oxidation states has demonstrated these compounds show unusual reactivity due to the presence of accessible frontier molecular orbitals with a small HOMO–LUMO gap.^28–31^ Accordingly, oxidative addition to the parent low-valent aluminium(i) reagent Al-2^32^ is calculated to be thermodynamically favourable (Scheme 2c). In the absence of a catalyst Al-2 does not react with a C–H bond of benzene.^33^ However, the group of Fischer reported that, when reacted with a Ni(0) precursor in C_6_H_6_, the related Al(i) reagent AlCp* readily inserts into the C–H bond of the solvent.^34^ Carbon–hydrogen bond functionalisation was proposed to take place *via* an initial oxidative addition to the transition metal centre followed by the migration of both the hydride and phenyl group to the appended aluminylene ligand. However, this reaction is not catalytic since the resulting H–Al–Ph moiety remains coordinated to the transition metal.

**Scheme 3:**
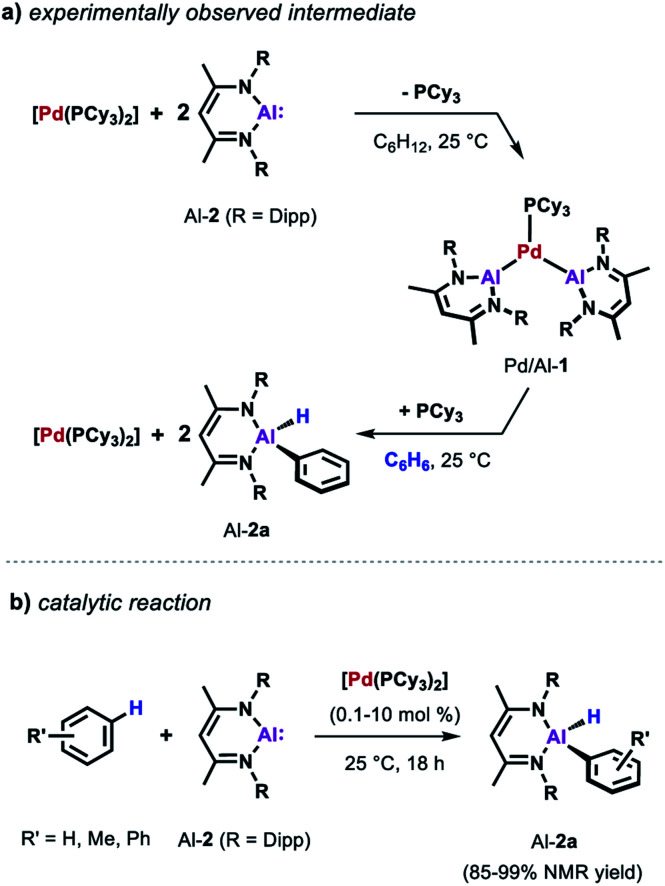
(a) Reaction of [Pd(PCy)_3_] with Al-2. (b) Catalytic alumination of benzene.

Similarly, the reactivity of Al-2 is significantly enhanced by the addition of [Pd(PCy_3_)_2_].^35^ The stoichiometric reaction of low-valent Al(i) complex Al-2 with the Pd(0) catalyst precursor in cyclohexane yields complex Pd/Al-1 in which two aluminylene ligands are bound to the transition metal centre (Scheme 3a). Formation of this complex could also be observed in benzene but it proved to be unstable as the reaction with the solvent resulted in the clean formation of the respective C–H alumination product as well as the regeneration of [Pd(PCy_3_)_2_]. The reaction could be run with [Pd(PCy_3_)_2_] as a catalyst and takes place at room temperature with loadings as low as 0.1 mol% (Scheme 3b). This is remarkable when compared with the rather harsh reaction conditions required for the C–H alumination of fluoroarenes and heteroarenes with the parent aluminium dihydride Al-1 reported before.

**Scheme 4:**
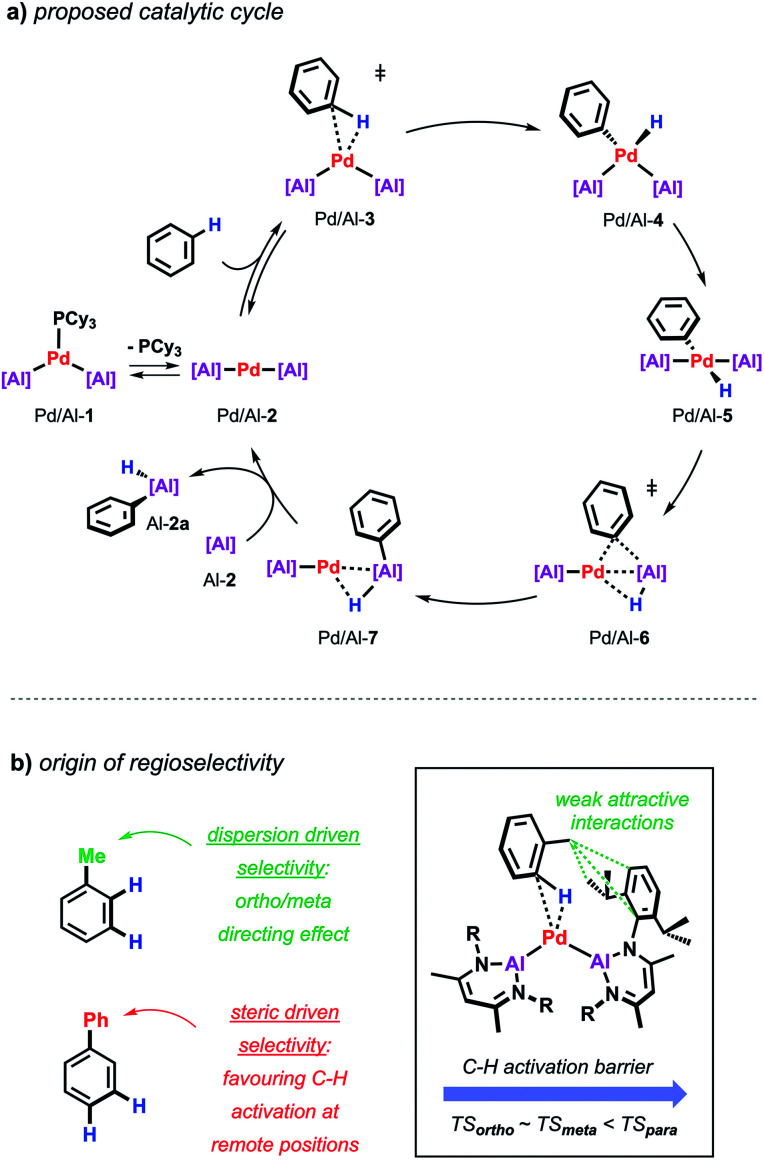
(a) Proposed catalytic cycle for the Pd-catalysed alumination of unfunctionalised arenes. (b) Origin of regioselectivity in Pd-catalysed alumination of arenes.

Further insight into the mechanism of the palladium catalysed alumination of unfunctionalised arenes with Al-2 could be obtained by means of DFT calculations (Scheme 4a). The calculations were initiated from Pd/Al-2 formed from dissociation of the PCy_3_ ligand from Pd/Al-1. Pd/Al-2 is a two-coordinate 14-electron fragment with a bent geometry. Association of benzene with this fragment can occur through formation of a weak encounter complex with the ligand sphere. C–H bond cleavage occurs by a classical three-centred oxidative addition transition state (Pd/Al-3) affording the *cis*-Pd(ii) intermediate Pd/Al-4. This is close in energy to its *trans*-isomer Pd/Al-5, from which the reaction proceeds *via* an unusual double migration step (Pd/Al-6) in which the Al–C and the Al–H bonds are formed in a concerted ligand transfer of both Ph and H from palladium to the aluminium centre to yield intermediate Pd/Al-7. This step proceeds with a two-electron transfer from the aluminium to the palladium centre (Pd(ii)/Al(i) to Pd(0)/Al(iii)). Exchange of the σ-bounded alane product by another aluminylene ligand finally closes the catalytic cycle. The calculated barriers as well as kinetic experiments suggest C–H bond cleavage at the Pd(0) centre is the turnover limiting step in the reaction.

During these studies an unusual mode of regiocontrol was observed for the Pd/Al system. The Pd-catalysed reaction of Al-2 with methyl-substituted arenes such as toluene or xylenes takes place primarily in the *ortho*- and *meta*-position which is complementary to traditional C–H borylation methodologies preferably affording the respective *meta*- and *para*-functionalised products.^36–38^ The origin of regioselectivity could be rationalised by the difference in energy of the transition states for the C–H bond activation step and can be traced back to non-covalent interactions between the methyl group of the arene and the aromatic substituents on the aluminylene ligands. In case of a phenyl substituent where attractive dispersive interactions are not expected, a switch of selectivity can be observed resulting in the activation of the remote *meta*/*para*-positions of the substrate probably controlled by steric effects as established for related C–H borylations (Scheme 4b).^39^

**Scheme 5:**
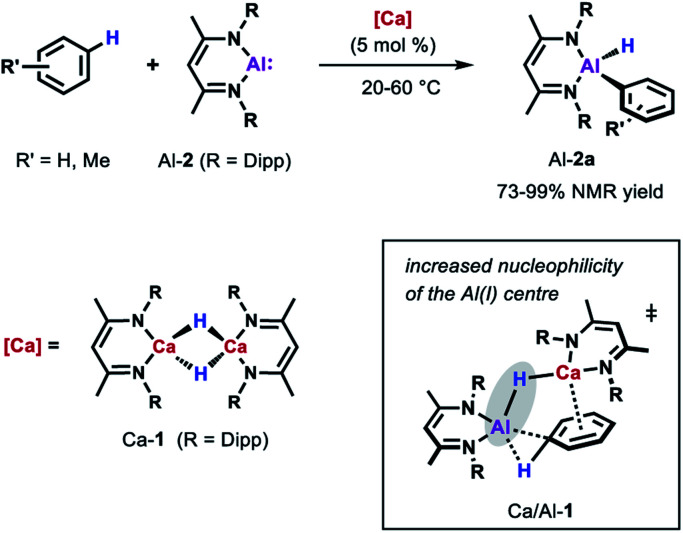
Ca-catalysed alumination of arenes.

More recently, the group of Harder showed that also catalytic amounts of a molecular calcium hydride complex can promote the oxidative addition of the sp^2^ C–H bonds of unactivated arenes (benzene, toluene and xylene) to Al-2 at room temperature (Scheme 5).^33^ In contrast to palladium catalysed reaction, C–H bond cleavage is thought to be facilitated by the combined action of a nucleophilic Al centre and arene activation by π-coordination to a Lewis acidic Ca centre. Similar to anionic alumanyl complexes that are capable of breaking strong C–H bond of benzene,^23,40,41^ an unusual low positive charge on Al could be identified through calculations on the relevant Al–H–Ca species Ca/Al-1. Remarkably, the Ca-catalysed alumination of toluene occurs almost exclusively at the *meta*-position of the substrate, consistent with a mechanism involving nucleophilic attack of the aluminium reagent on an arene with an electron-donating substituent.^42,43^

**Scheme 6:**
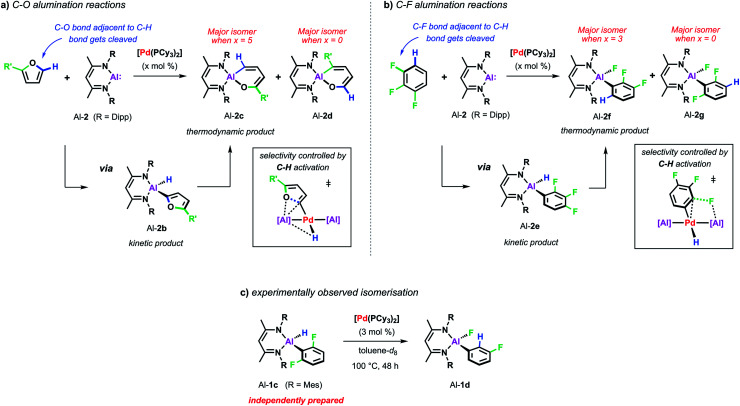
(a) Pd-catalysed C–O alumination of furans. (b) Pd-catalysed C–F alumination of fluoroarenes. (c) Pd-catalysed isomerisation from Al-1c to Al-1d.

Recent studies have revealed that the C–H activation step also plays an important role in reactions that break strong C–O and C–F bonds. In the presence of palladium catalyst, C–O and C–F alumination reactions with Al-2 can also occur *via* a C–H activation process. This step has an impact on the scope and selectivity of the transformations as C–H bonds act as a transient directing group, being broken and remade along the reaction pathway (Scheme 6).^44,45^

For example, the non-catalysed reaction of Al-2 with 2-methylfuran at 80 °C affords two isomers (Al-2c and Al-2d) of the ring-expanded metallacycle (Scheme 6a). The major species (Al-2d) results from insertion into the more hindered sp^2^ C–O bond. DFT calculations suggest a mechanism proceeding *via* an initial (4 + 1) cycloaddition of Al-2 and the furan followed by the rearrangement of the resulting bicyclic intermediate. Selectivity originates from the latter step and can be traced back to the electronic influence of the substituent weakening the adjacent sp^2^ C–O bond. Higher and complementary selectivity is obtained in the presence of catalytic [Pd(PCy_3_)_2_]. At room temperature, C–H alumination at the 2-position of the substrate affords a kinetic product (Al-2b) which, under more forcing conditions, converts into the thermodynamic ring-expanded aluminium cycle Al-2c (Scheme 6a). In contrast to the non-catalysed reaction, C–O bond alumination occurs exclusively at the reaction site adjacent to the intermediately activated C–H position. Mechanistic studies revealed that not only the C–H alumination step but also the rearrangement of the kinetic into the thermodynamic product is promoted by the palladium catalyst. DFT calculations suggested that the C–H aluminated intermediate can be re-activated at palladium following an alternative high energy pathway that leads to the thermodynamic product. As the key step in this mechanism, C–O bond cleavage involves the attack of an aluminium based metalloligand on the 2-palladated heterocycle. The C–H bond guides the catalyst to the adjacent site by being broken and remade along the reaction pathway, this mechanism explains the unusually high selectivity of the palladium catalysed reaction. The catalytic approach allows expansion of the substrate scope beyond furans to 2,3-dihydrofuran and 3,4-dihydropyran which do not react cleanly with Al-2 in absence of [Pd(PCy_3_)_2_].

A role for reversible breaking of a C–H bond could also be identified for the C–F alumination reaction of fluoroarenes with Al-2 (Scheme 6b). In absence of [Pd(PCy_3_)_2_], the aluminium(i) reagent does not show any appreciable reactivity towards low-fluorine-content substrates such as 1,2,3-trifluorobenzene at room temperature.^46^ At higher temperatures, the non-catalysed reaction of this substrate affords the oxidative addition product in moderate yield and as a mixture of two regioisomers (Al-2f and Al-2g). The catalytic approach instead allows the C–F alumination of mono-, di- or trifluorobenzenes to take place under exceptionally mild conditions and with high selectivity. For example, while the major product of the non-catalysed reaction of 1,2,3-trifluorobenzene results mainly from reaction of the central C–F bond (Al-2g), the complementary regioisomer (Al-2f), in which the aluminium fragment is installed next to an existing C–H bond, is preferably formed under catalytic conditions. Mechanistic studies in combination with DFT calculations suggest a stepwise C–H to C–F functionalisation process which leads the catalyst to a C–F bond adjacent to a reactive C–H site and thus accounts for the observed regioselectivity. Experimental support for C–H functionalisation playing a role in catalysis was obtained by the identification of a 100% atom efficient palladium catalysed isomerisation of the kinetic C–H alumination product Al-1c to the thermodynamic C–F alumination product Al-1d (Scheme 6c).

**Scheme 7:**
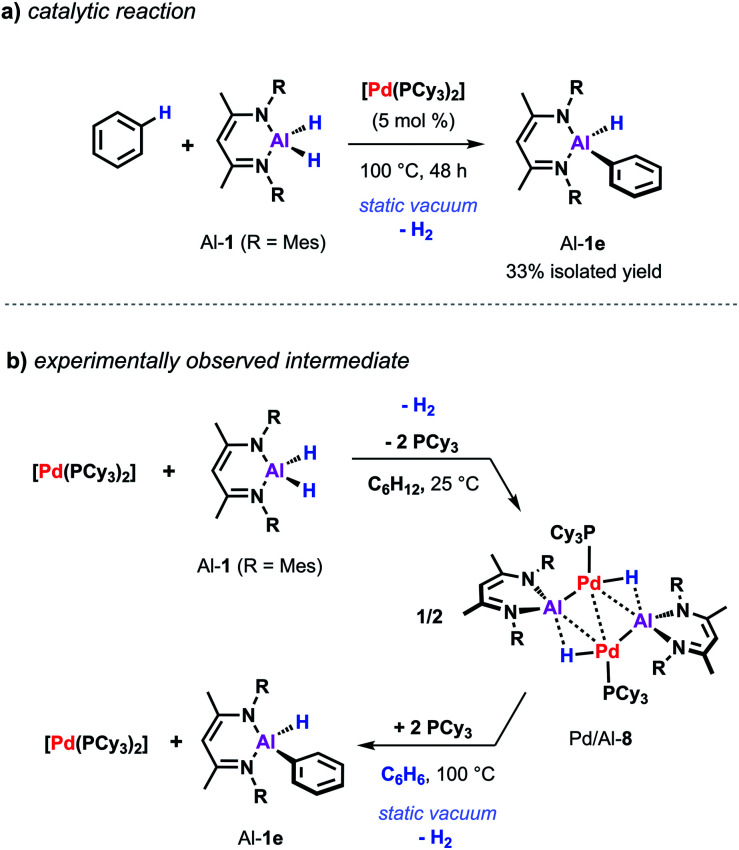
(a) Pd-catalysed C–H alumination of benzene with Al-1. (b) Stoichiometric reactivity of [Pd(PCy_3_)_2_] with Al-1.

Despite the promising progress in the catalytic C–H metalation using low-valent aluminium reagent Al-2 the implementation of the respective hydride species (Al-1) is still desirable due to their higher stability and more convenient and efficient preparation. Although being endergonic based on DFT calculations (Scheme 2c) catalytic C–H alumination of benzene with Al-1 could be realised experimentally by conducting the reaction under static vacuum as removal of the liberated H_2_ permits catalytic turnover (Scheme 7a).^35^ The same approach has been successfully applied on the C–O and C–F alumination of furans and fluoroarenes respectively.^44,45^

**Scheme 8:**
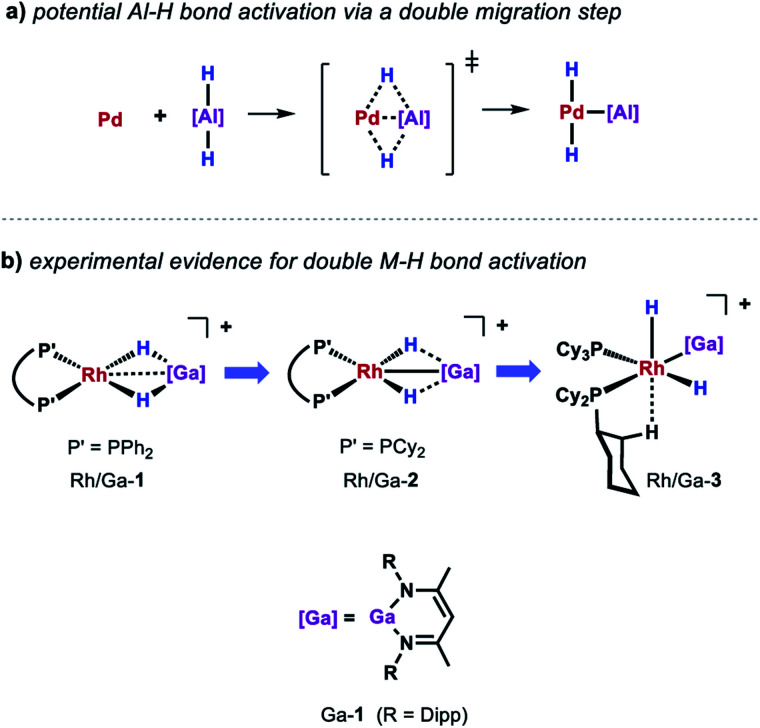
(a) Potential pathway for the activation of Al-1 at Pd(0). (b) Experimental evidence for a double migration step through structural snapshots of the progressing Ga–H bond activation at Rh(i).

While a consistent mechanistic picture could be established for the alumination of sp^2^ C–H bonds with Al-2, it remains unclear if and how palladium aluminylene intermediates are formed when using Al-1 as a stoichiometric reagent.^47^ For example, one might imagine an elementary step that takes place in a similar but reverse fashion to the aforementioned double migration step from Pd/Al-5 (Scheme 8a). Experimental evidence for such a concerted double H–M–H bond activation at a transition metal centre has recently been provided by Aldridge and co-workers demonstrating that the analogous gallium dihydride is prone to react with a Rh(i) precursor to form the respective Rh(iii) dihydride Rh/Ga-3 featuring a neutral Ga(i) gallylene ligand (Scheme 8b).^48^ An alternative metal-mediated dehydrogenation of gallium dihydride was reported by the same group and involves the oxidative addition of Ga–H to metal carbonyl complexes followed by subsequent loss of H_2_ under photochemical conditions.^49^

The equivalent process in the Pd/Al system is yet to be observed. Nevertheless, stoichiometric experiments unveiled Al–H bond activation and partial dehydrogenation of the aluminium dihydride when reacted with diverse palladium precursor complexes.^47^ For example, room temperature reaction of the aluminium dihydride Al-1 with [Pd(PCy_3_)_2_] led to the isolation of the tetrametallic complex Pd/Al-8 (Scheme 7b).^35^ The position and number of the hydride ligands in Pd/Al-8 were confirmed by single crystal neutron diffraction studies of an analogue complex featuring 2,6-xylyl substituents on the metalloligand.^47^ Formation of this complex involves partial dehydrogenation of Al-1 and proceeds with liberation of H_2_. Heating Pd/Al-8 in benzene under static vacuum results in the regeneration of [Pd(PCy_3_)_2_] accompanied by C–H alumination of the solvent. Nevertheless, it remains unclear at the moment if Pd/Al-8 constitutes an on-cycle intermediate in dehydrogenative alumination reactions.

### C–H magnesiation

**Scheme 9:**
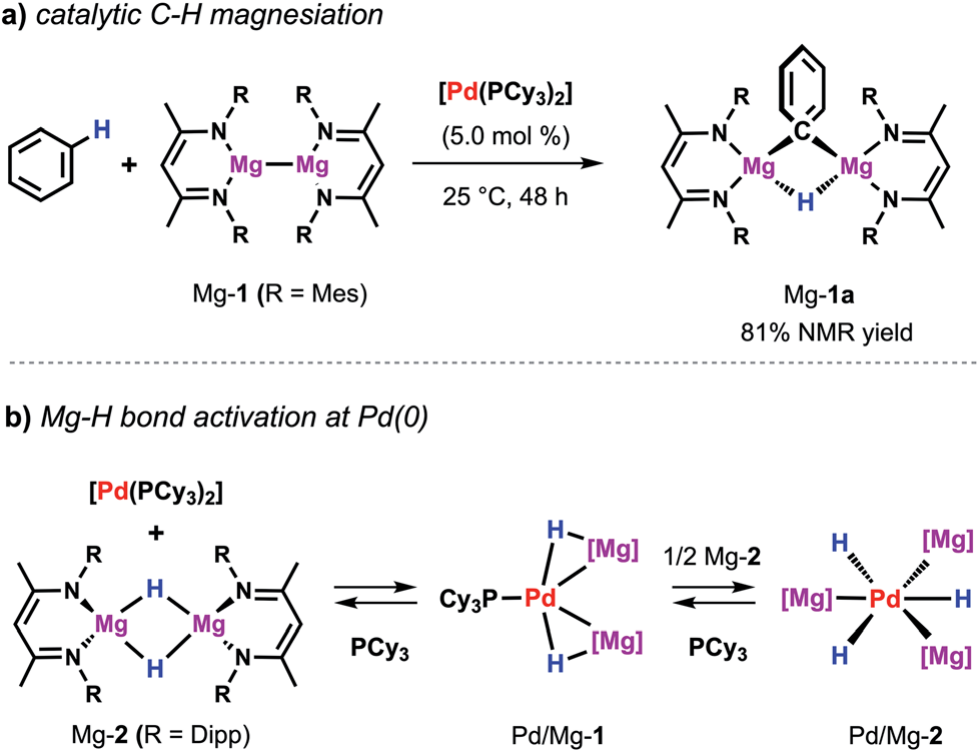
(a) Pd-catalysed magnesiation of benzene. (b) Stoichiometric reactivity of [Pd(PCy_3_)_2_] with Mg-2.

The same palladium diphosphine complex also proved effective in the catalytic reaction of a low-valent magnesium complex with C–H bonds.^50^ The reaction of Mg-1 with benzene in presence of catalytic [Pd(PCy_3_)_2_] results in the formation of the kinetically stabilised organomagnesium hydride Mg-1a (Scheme 9a). In the solid state, this species exists as a dimer in which two equivalent magnesium sites are bridged by 3-centre, 2-electron Mg–H–Mg and Mg–C–Mg bonds. The reaction takes place at room temperature whereas, even at high temperatures, no reaction is observed in the absence of a catalyst. While Mg-1 and related species are emerging as versatile reagents in synthesis,^22,51^ this is the first example of their use in the activation of inert carbon–hydrogen bonds. Preliminary kinetics experiments suggest that breaking of the C–H bond is not likely to be the turnover limiting step. The value of KIE obtained of 1.1 contrasts that reported for the C–H alumination of benzene with low-valent aluminium complexes described above which showed a large primary KIE of 5.8 ± 0.1.^35^ These findings indicate that magnesiation of benzene may be mechanistically distinct from a related reaction involving the low-valent aluminium reagent Al-2.

Dehydrogenative magnesiation of benzene using the parent dimeric magnesium hydride complex (Mg-2) did not lead to the clean formation of Mg-1a. Nevertheless, stoichiometric reactions of Mg-2 with [Pd(PCy_3_)_2_] revealed the reversible cleavage of the Mg–H bond at the transition metal centre (Scheme 9b).^52^ Stepwise substitution of the phosphine ligands could be observed in solution leading to an equilibrium between the Pd(0) precursor as well as the two new bimetallic hydride complexes Pd/Mg-1 and Pd/Mg-2 mapping the progressing Mg–H bond activation process. Near complete cleavage of the magnesium hydride bonds occurs in Pd/Mg-2 leading to the formation of pairs of σ-donor (H^−^) and σ-acceptor (Mg^+^) ligands at a Pd(0) centre adopting an unusual hexagonal planar coordination geometry. The heterometallic hydride complexes undergo H/D-exchange in benzene-d_6_ demonstrating that these species are essentially competent for C–H activation reactions.

## Perspective

In this mini-review, we summarised recent efforts to extend the concept of established C–H borylation methodologies to C–H metalation reactions that allow the direct functionalisation of aromatic sp^2^ C–H bonds into C–Al and C–Mg bonds in a straightforward and atom efficient way. These new reactions currently rely on the implementation of a palladium catalyst. Mechanistic studies disclosed new reaction pathways in which heterometallic intermediates play a central role. We believe that these first examples are just the beginning of a new chapter in C–H activation chemistry.

Future efforts will undeniably focus on the diversification of the new catalytic transformations. These may comprise an expansion of the substrate scope from aromatic and heteroaromatic compounds to other hydrocarbons including the functionalisation of sp^3^ C–H bonds. Moreover, mechanistic understanding as well as the knowledge about the factors that determine reactivity and selectivity can be exploited on a general basis and used for rational design of novel catalytic systems. As exemplified by the calcium hydride mediated C–H alumination discovered by the group of Harder, the use of precious metals such as palladium is not a prerequisite to overcome the kinetic barriers in these reactions. This approach could lead to new developments in the growing field of base metal catalysis.

In order to exploit the synthetic potential of these organometallated reagents, the development of efficient derivatisation methods has to be considered. Transition metal catalysed cross-coupling reactions are likely to play a central role in this respect. Organozinc and -magnesium compounds are widely used reagents in Negishi- and Kumada-type C–C cross-coupling protocols. As these reactions occur in presence of the same palladium pre-catalyst, one might envision two step processes that combine the C–H activation and C–C bond formation steps in a one-pot reaction. Especially in view of widely employed Negishi cross-coupling reactions, an expansion of the current alumination and magnesiation reactions to the direct formation of C–Zn bonds might largely increase the applicability of these new C–H metalation methodologies. On the other hand, the direct access to C–Al derivatives provides an entirely new perspective in this field and may potentially lead to the development of a new class of transition metal catalysed coupling reactions.

In the light of ongoing attempts tending to improve the scope, efficiency and selectivity of C–H borylation catalysts we described how some of these challenges may be addressed by translating the established concept into new methodologies. The catalytic C–H metalation processes presented herein clearly differ from conventional borylation reactions. The new reaction pathways give rise to complementary reactivity and selectivity as illustrated by the distinct site selectivity for toluene and xylenes or the ring opening of heterocycles proceeding with predictable selectivity. Finally, the more pronounced polarity of C–M bonds in the resulting organometallic reagents will inevitably provide new opportunities to organic synthesis reaching beyond the widespread application of established boronate esters.

## Conflicts of interest

There are no conflicts to declare.

## Supplementary Material
